# Identification of winter wheat pests and diseases based on improved convolutional neural network

**DOI:** 10.1515/biol-2022-0632

**Published:** 2023-07-06

**Authors:** Jianbin Yao, Jianhua Liu, Yingna Zhang, Hansheng Wang

**Affiliations:** School of Information Engineering, North China University of Water Resources and Electric Power, Zhengzhou, 450046, China

**Keywords:** wheat diseases and pests, data expansion, transfer learning, mixed attention mechanism

## Abstract

Wheat pests and diseases are one of the main factors affecting wheat yield. According to the characteristics of four common pests and diseases, an identification method based on improved convolution neural network is proposed. VGGNet16 is selected as the basic network model, but the problem of small dataset size is common in specific fields such as smart agriculture, which limits the research and application of artificial intelligence methods based on deep learning technology in the field. Data expansion and transfer learning technology are introduced to improve the training mode, and then attention mechanism is introduced for further improvement. The experimental results show that the transfer learning scheme of fine-tuning source model is better than that of freezing source model, and the VGGNet16 based on fine-tuning all layers has the best recognition effect, with an accuracy of 96.02%. The CBAM-VGGNet16 and NLCBAM-VGGNet16 models are designed and implemented. The experimental results show that the recognition accuracy of the test set of CBAM-VGGNet16 and NLCBAM-VGGNet16 is higher than that of VGGNet16. The recognition accuracy of CBAM-VGGNet16 and NLCBAM-VGGNet16 is 96.60 and 97.57%, respectively, achieving high precision recognition of common pests and diseases of winter wheat.

## 1 Introduction

Wheat is one of the main food crops in China, and pests and diseases are a major problem in wheat production that seriously affects the yield and quality of wheat [1,2]. According to statistics, the planting area of wheat in China was as high as 23.57 million hm^2^ in 2021, and the annual output was 13.695 million tons. It is estimated that the occurrence area of wheat pests and diseases will reach 54 million hm^2^ in 2022. The overall situation is biased, including 27.3 million hm^2^ diseases and 26.7 million hm^2^ pests.

Traditional wheat pest identification mainly relies on staff patrols or the machine vision technology for auxiliary identification. Machine vision technology is an efficient method for automatic detection of crop pests and diseases, and its core is image processing. Currently, there is no general separation theory in image processing, resulting in insufficient scalability of the algorithm. In addition, the use of machine vision technology to identify winter wheat pests and diseases still has problems, such as complex treatment process, high labor cost, strong subjectivity, and difficulty in timely detection of large-scale infection of pests and diseases. In recent years, with the continuous development of artificial intelligence technology, deep learning [3] has gradually replaced machine learning and become the main representative of artificial intelligence technology, and has gradually been applied to the identification of crop pests and diseases.

As a representative model of deep learning, convolutional neural network (CNN) can automatically extract the features of diseases and pests, simplify the identification process of winter wheat pests and diseases, reduce labor costs, greatly improve the accuracy and stability of pests and diseases identification, and improve the identification efficiency. It can effectively promote the informatization and intelligence of agriculture, which is of great significance for the stable development of agriculture. For the identification of tomato pests and diseases, Fuentes et al. [4] added various types of refined filter groups on the traditional CNN to solve the problem of false alarm of the boundary box generator during the training process, as well as the problem of small sample number and unbalanced distribution of species, improving the identification accuracy to 96%. Yadav et al. [5] proposed a CNN model based on imaging methods, which realized the detection of bacterial spot disease of peach leaf, with a recognition accuracy of 98.75%. For small samples of hop pests and diseases, Lu and Chen improved the deep residual network (ResNet) model based on the attention mechanism, and the recognition accuracy of the improved model reached 93.27% [6]. Xiang et al. [7] proposed a plant pests and diseases identification method based on Xception model, which combined the multi-scale convolution and group convolution and introduced the dense connection mode to improve feature reuse between feature maps. The comprehensive accuracy of this method was 91.90%.

According to the research status of deep learning in the identification of pests and diseases, most of them mainly focus on large-leaf plants (tomatoes, cotton, grapes, and more), while that for small-leaf plants is relatively rare. To this end, taking the winter wheat as an object, a standardized dataset for the four common pest and disease samples collected in the natural environment of the test field is built. A traditional CNN model for the identification of winter wheat pests and diseases is constructed, and the migration learning technology and attention mechanism are introduced for improvement. Finally, the accurate identification of winter wheat common pests and diseases in the natural environment is realized.

## 2 Data and methods

### 2.1 Datasets

The dataset comes from the agricultural and water teaching practice base of North China University of Water Resources and Electric power, and is collected in the natural environment. The winter wheat variety is Xinhuamai 818, a new variety developed by Henan Agricultural University in 2018. The standardization of datasets includes sample sorting, preprocessing, and data enhancement.

**Figure 1:**
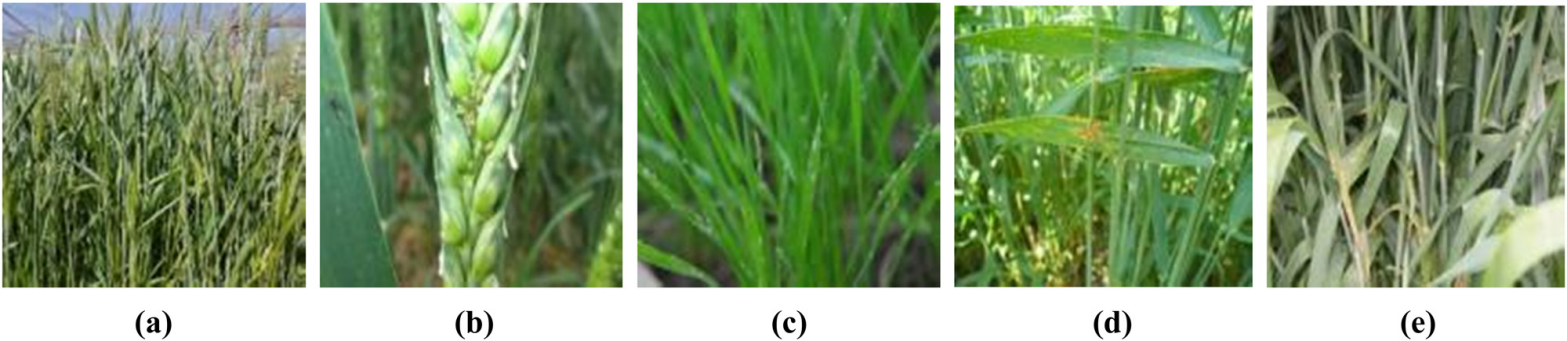
Wheat samples: (a) healthy wheat; (b) aphids; (c) powdery mildew; (d) leaf rust; and (e) stripe rust.

**Figure 2:**
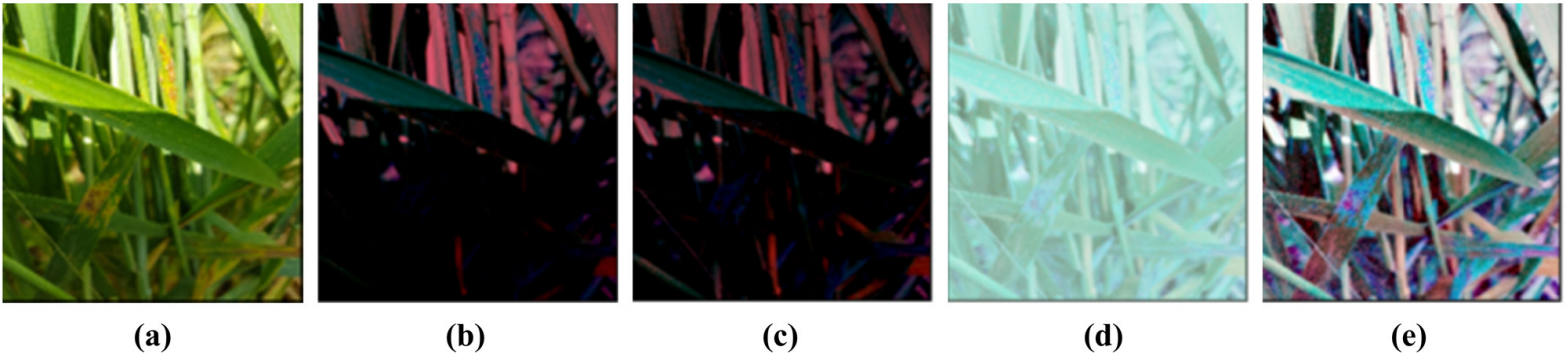
Image processing effects of different Retinex series algorithms: (a) original picture, (b) SSR, (c) MSR, (d) MSRCR, and (e) AutoMSRCR.

1) Dataset sorting and division. The dataset contains five categories: healthy wheat, aphid wheat, powdery mildew wheat, leaf rust wheat, and stripe rust wheat. The dataset contains 1,003 samples from different periods of wheat growth. Some samples are shown in Figure 1.
  According to the most common sample division ratio (8:2), each type of dataset is randomly divided into training sets and test sets. The composition of the datasets is shown in Table 1.
2) Preprocessing. To meet the input sample size requirements of the CNN model, the nearest neighbor image interpolation method is used to uniformly process the original image to two specifications of 224 × 224 (pixels) and 227 × 227 (pixels) images. Normalization processing refers to obtaining the mean values of all samples in the dataset on the red, green, and blue channels separately, and then subtracting the mean values of each channel from each sample to obtain the standardized image. Normalization of images can effectively avoid gradient explosion during training.
3) Data enhancement. The pre-processed image is enhanced to reduce the light interference. The image enhancement method based on scientific experiments and analysis, Retinex series algorithm, is selected to enhance the image. Figure 2 shows the enhancement of single scale retinex (SSR) [8], multi-scale retinex (MSR) [9], multi-scale retinex with color restoration (MSRCR) [10], and auto multi-scale retinex with color restoration (AutoMSRCR) [11] algorithms.

**Table 1:** Composition of winter wheat dataset

Categories	Sample number/piece		
	Training sets	Test sets	Total
Aphids	36	10	46
Powdery mildew	58	15	73
Healthy winter wheat	113	30	143
Leaf rust	289	74	363
Stripe rust	301	77	378
Total	797	*206*	1,003

It can be seen from Figure 2 that the images enhanced by SSR and MSR algorithms are too dark, and a lot of image details will be lost. The image processed using the default parameters of the MSRCR algorithm are also not optimal. The AutoMSRCR algorithm is thus selected to enhance the samples.

### 2.2 CNNs

Considering the environmental conditions of hardware equipment, the following three convolutional neural network models with low requirements for computer hardware and excellent image classification effect are selected to realize the classification and recognition training of common pest and disease images of winter wheat.

1) AlexNet network model, which won the championship in the 2012 image recognition contest with 57.1% accuracy and 80.2% top-5 recognition rate, has established the core position of CNN in image classification algorithm [12]. The model consists of five convolution layers and three full connection layers. ReLu activation function layer is set after the convolution layer and full connection layer, and LRN is set after the first two full connection layers, which is conducive to rapid convergence and generalization enhancement of the model. Zhou et al. [13] proposed a new method that uses AlexNet with ImageNet transfer learning as the feature extractor and optimized and regularized extreme learning as the classifier. The test outcome illustrates that on some apparel classification with style datasets, the precision, recall, *F*1-score, and accuracy of the proposed algorithm are 93.06, 93.17, 92.82, and 93.14%, respectively. The results verify that the raised algorithm significantly ameliorates the classification property of clothing image algorithms.
2) VGGNet [14] network model is a series of neural network models proposed by the VGG experimental group of Oxford University, and has many variants. The most popular one is the VGGNet 16 network model, whose accuracy rate 92.3% on ImageNet ranks the top list 5. The VGGNet16 network model uses a smaller convolution kernel for feature extraction, including 13 volumelayers and 3 full connection layers. Also, these models, especially VGGNet 19 [15].
3) Inception-V3 [16] network model. Such a series of networkmodels were proposed by the Google team in 2014. Before that, the CNN models were all based on stacking convolution layers to extract features. The Inception networkmodel indicativelyput forward the Inception module. According to various structures, the Inceis divided into multiple versions, hereof the Inception-V3 model is utilized to the most. Also, other CNNs, particularly ResNet18 [17], as well ResNet50 [18] improving.

### 2.3 Data expansion technology

Due to the problem of small dataset size and uneven distribution of sample sizes among different types in the manually collected basic dataset in this article, this section introduces data augmentation technology to expand the basic dataset, mainly using the following methods.

1) Add random noise and random filtering. The dataset in this article was collected in the natural environment of the experimental field, so the background is relatively complex and there is also a lot of noise and light wave interference. Adding random noise and random filtering can reduce the interference of complex backgrounds on training results and better improve the generalization ability of the model. In this work, several common filters are selected, and random numbers are generated to make random selection among median filter, Gaussian blur filter, and other types.
2) Perform random rotation and random offset. Due to the lack of unified regulations on the target position during the sampling process, and the existence of multi-angle issues such as positive and negative, far and near, this article randomly rotates and offsets the samples to improve the model’s generalization ability to target position changes.
3) Add random color jitter. The collection of samples in the afternoon, on both sunny and cloudy days, will have significant differences in color. Therefore, random color jitter will be added to improve the model’s generalization ability to lighting factors. This article expands the sample by generating random numbers to randomly select from several changes in image saturation, brightness, contrast, and sharpness.

### 2.4 Transfer learning technology

Due to the small size of the dataset, it cannot meet the training of large network models, and the transfer learning technology [19] is introduced to improve the recognition of the model. Transfer learning can be divided into freezing source model and fine-tuning source model.

1) Freezing source model refers to freezing the parameters of other layers of the source model during training, and modifying and training only the parameters of the last fully connected layer according to the classification number of the new dataset [20]. At this time, the source model is equivalent to a feature extractor.
2) Fine-tuning source model refers to fine-tuning the parameters of the source model during training. According to different fine-tuning layers, the fine-tuning source model can be divided into fine-tuning part layers and fine-tuning all layers [21].

### 2.5 Mixed attention mechanism

**Figure 3:**
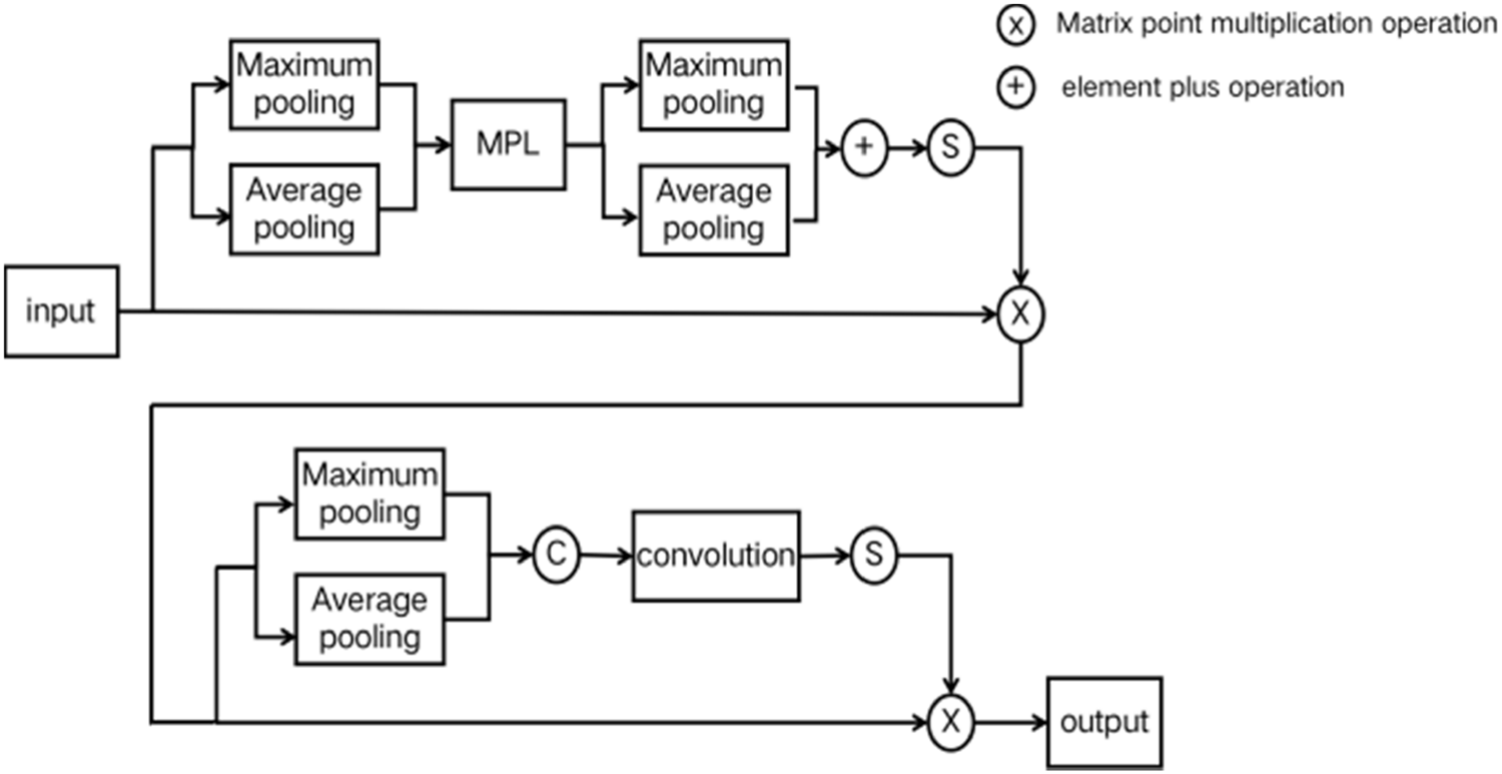
Structural diagram of CBAM module.

CBAM module is a mixed attention module proposed by Sanghyun Woo et al. in 2018. This module integrates the channel attention mechanism and spatial attention mechanism, and gives different weights to each channel and each position of the feature map, respectively. In the channel domain, it enhances relevant channels and suppresses irrelevant channels; in the spatial domain, it filters out useful features and suppresses useless features. Its structure is shown in Figure 3.

**Figure 4:**
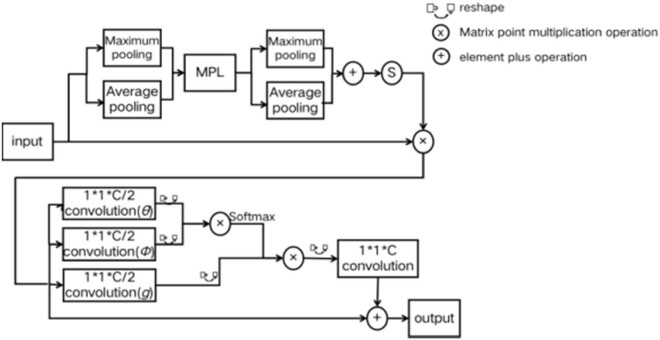
Structural diagram of NLCBAM module.

It can be seen from Figure 3 that the spatial attention module of CBAM uses convolution to fuse the pooled feature maps. Due to the limitations of convolution, this module can only achieve the correlation between local regions, which limits the correlation between different locations with a certain distance. This work replaces the spatial attention module of CBAM module with the non-local attention mechanism of non-local module, to propose the NLCBAM module. On the basis of CBAM, it breaks through the limitation that convolution can only consider local regions and increases the receptive field of the network. The structure is shown in Figure 4.

### 2.6 Model evaluation index

The accuracy, precision rate of each classification, recall rate, and {F}_{\text{1}}] are used to evaluate the model. The calculation method of each index is as follows:

1) Accuracy ( {A}_{\text{1}}] ) represents the proportion of correctly classified samples in the total samples of the dataset, and is calculated as follows:
(1) {A}_{1}=\frac{\mathop{\sum }\limits_{i=1}^{N}(\text{TP}+\text{TN})}{\text{ALL}},]
  where, *N* is the category of datasets, *N* = 5, \text{ALL}] represents the total number of samples in the dataset, \text{TP}] represents the number of positive samples classified as positive samples in this category, and \text{TN}] represents the number of negative samples classified as negative samples.
2) The precision rate ( {P}_{\text{1}}] ) represents the proportion of the actual positive samples among the samples predicted to be positive samples in each category, and is calculated as follows:
(2) {P}_{1}=\frac{\text{TP}}{\text{TP}+\text{FP}},]
  where FP represents the number of negative samples classified as positive samples in this category.
3) The recall rate ( {R}_{\text{1}}] ) represents the proportion of positive samples predicted to be positive samples in each category, and is calculated as follows:
(3) {R}_{\text{1}}=\frac{\text{TP}}{\text{TP}+\text{FN}},]
  where FN represents the number of positive samples classified as negative samples in this category.
4) *F*1 ( {F}_{\text{1}}] ) is the average of the precision rate and recall rate, which is a comprehensive index, and is calculated as follows:

(4) {F}_{\text{1}}=\frac{2\hspace{.25em}\ast \hspace{.25em}\text{Precision}\hspace{.25em}\ast \hspace{.25em}\text{Recall}}{\text{Precision}+\text{Recall}}.]

### 2.7 Test environment

The test machine is MacBook Air, with a CPU 1.6 GHz dual-core Intel Core i5, a memory of 8 GB, and a hard disk capacity of 256 GB. The operating system is macOS Monterey; the deep learning framework is Tensor Flow-CPU; the programming language is Python with 3.7.9 version, and the editor is PyCharm; the image processing library is cv2 and PIL, and the environment management software is Anaconda3.

## 3 Results analysis

### 3.1 Basic network selection

Based on the normalized dataset of winter wheat pests and diseases, this section realizes the identification of pests and diseases using AlexNet, VGGNet16, and Inception-V3 traditional CNNs. The models with the highest accuracy in all iterations of each network are selected as the final model of each network. To improve the training speed of the model, the training sets are trained in batches. The corresponding hyper parameters of each network model are shown in Table 2, and the test set evaluation indicators of each model are shown in Table 3.

**Table 2:** Hyper parameters corresponding to each network model

Parameters	AlexNet	VGGNet16	Inception-V3
Iterations/time	50	50	500
Number of samples per batch/piece	32	32	100
Loss function	Softmax cross entropy	Softmax cross entropy	Softmax cross entropy
Optimizer	Adam optimizer	Adam optimizer	Gradient descent optimizer
Learning rate	Attenuation learning rate	Attenuation learning rate	Constant learning rate

**Table 3:** Test set evaluation index of each network model

Categories	AlexNet			VGGNet16			Inception-V3		
	*P*_1_	*R*_1_	*F*_1_	*P*_1_	*R*_1_	*F*_1_	*P*_1_	*R*_1_	*F*_1_
Aphids	0.0000	0.0000	0.0000	0.0000	0.0000	0.0000	0.0000	0.0000	0.0000
Powdery mildew	0.2936	0.2625	0.2772	0.3315	0.2042	0.2527	0.3045	0.2032	0.2437
Healthy wheat	0.4352	0.5533	0.4872	0.8324	0.6618	0.7362	0.6583	0.7364	0.6952
Leaf rust	0.6473	0.6021	0.6239	0.7784	0.7435	0.7605	0.5932	0.6471	0.6189
Stripe rust	0.6483	0.7227	0.6835	0.6463	0.8829	0.7463	0.6364	0.6139	0.6249
Average	0.4049	0.4281	0.4143	0.5177	0.4981	0.4992	0.4385	0.4401	0.4366
*A*_1_	0.5827			0.6914			0.6037		

The parameters of Adam optimizer in the Table are set as beta_1 = 0.9 and beta_2 = 0.99. The initial learning rate of the attenuation learning rate method is set to 0.01, and the attenuation is 1/2 every 10 iterations. The learning rate of the constant learning rate method is always 0.01.

From Table 3, we can get the following four results: (1) The accuracy of the test set of AlexNet network model is 58.27%, and its recognition effect on stripe rust is the best. When *F*1 is 0.6835, it has the worst recognition on aphids; when *F*1 is 0, the recognition accuracy of powdery mildew is also low; when *F*1is 0.2772, the recognition accuracy is poor. (2) The accuracy of the test set of VGGNet16 model is 69.14%, and the recognition effect on leaf rust is the best. When *F*1 is 0.7605, the recognition effect on aphids is as poor as that of AlexNet model, and that on powdery mildew is also low, with *F*1 being only 0.2527. The effect of VGGNet16 model is better than AlexNet model. (3) The accuracy of Inception-V3 model is 60.37%. Its recognition effect on healthy wheat is the best, and *F*1 is 0.6952. The recognition effect on aphids is as poor as that of AlexNet model, and that on powdery mildew is also low, with *F*1 being only 0.2437. (4) In the three network models, VGGNet16 has the highest accuracy and is selected as the basic network.

### 3.2 Improvement based on data expansion

**Figure 5:**
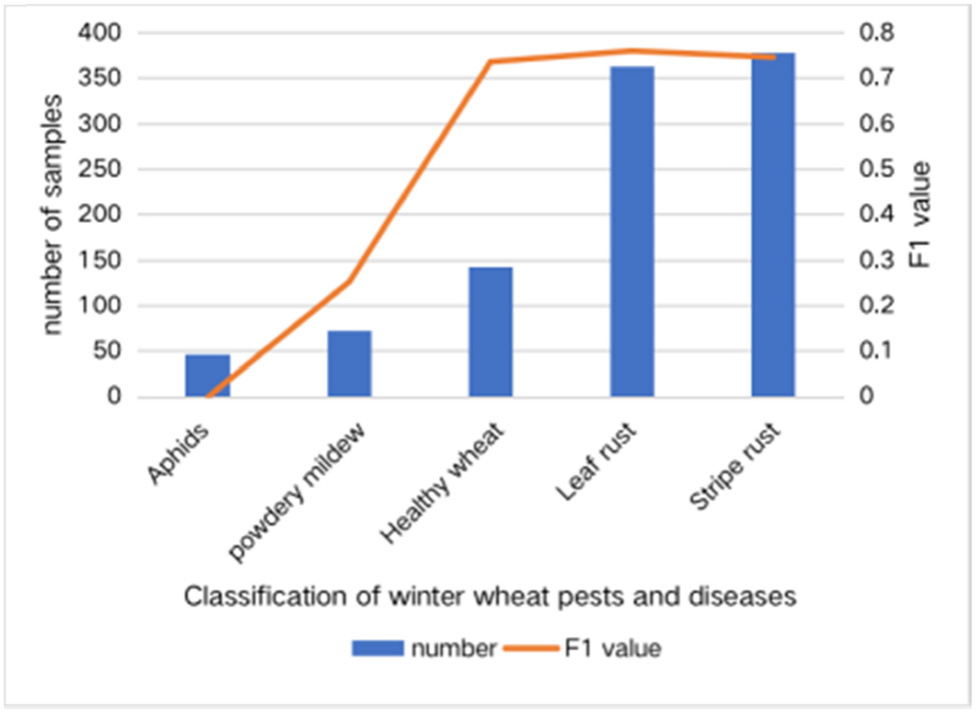
Number and *F*1 value of samples in each category of VGGNet16 final model.

From the experimental results in the previous section, it can be seen that the *F*1 of aphids and powdery mildew is relatively low. Combined with the number of samples in each category, it is found that the recognition effect is positively correlated with the number of samples, as shown in Figure 5.

It can be seen from Figure 5 that the low recognition rate of individual types is caused by the small amount of data of this type and the uneven distribution of the number of each type of dataset. To solve these problems, the training dataset is randomly expanded by adding random noise and random filtering, random rotation and random shift, and random color jitter.

To ensure that the proportion of each sample in each category is consistent, and that the number of samples in each category after expansion is relatively balanced, this study randomly expands all samples in each category by multiple. The composition of the expanded dataset is shown in Table 4.

**Table 4:** Composition of the expanded winter wheat pests and diseases dataset

Categories	Training set			Test set/piece
	Original data number/piece	Random expansion times/times	Data number after expansion/piece	
Healthy wheat	113	7	791	30
Stripe rust	301	3	903	77
Leaf rust	289	3	867	74
Powdery mildew	58	14	812	15
Aphids	36	22	792	10
Total	797		4,165	206

The model is trained based on the expanded training set and the hyper-parameters corresponding to the VGGNet16 model in Table 2. The model evaluation indicators are shown in Table 5.

**Table 5:** Evaluation index of VGGNet16 test set after expansion

Categories	*P*_1_	*R*_1_	*F*_1_
Aphids	0.3641	0.4436	0.3999
Powdery mildew	0.4428	0.2872	0.3484
Healthy wheat	0.7394	0.7422	0.7408
Leaf rust	0.6435	0.7048	0.6728
Stripe rust	0.7471	0.7374	0.7422
Average	0.5874	0.5830	0.5808
*A*_1_	0.6837		

Table 5 shows that the accuracy of VGGNet16 model based on the expanded dataset is 68.37%. Compared with the results of the original data in Table 3, the recognition effect of the model on aphids has been significantly improved, and *F*1 changes from 0.0 to 0.3999. *F*1 of powdery mildew changes from 0.2527 to 0.3484, which is increased by 0.0957. This shows that the expanded training set samples can well solve the problem of low *F*1 recognition of aphids and powdery mildew using this model.

### 3.3 Improvement based on transfer learning

**Figure 6:**
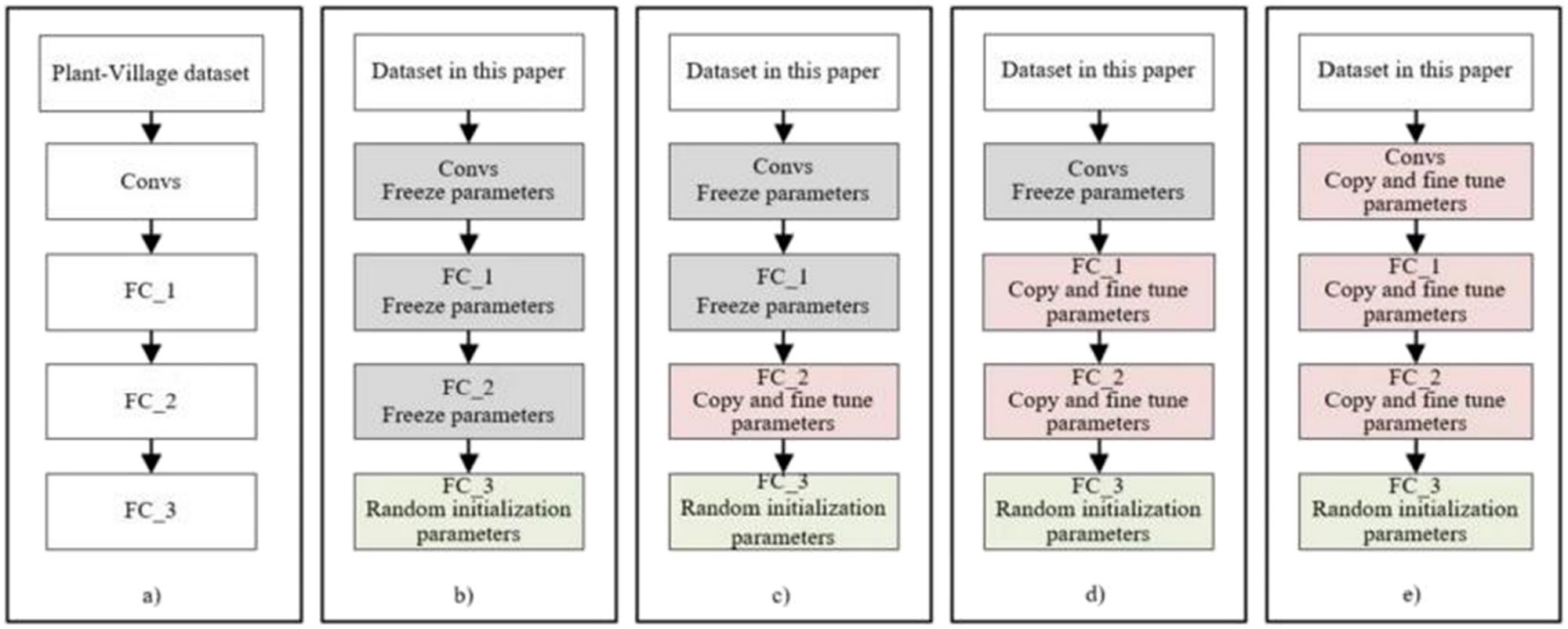
Experimental scheme based on transfer learning. (a) VGGNet16, (b) Experiment 1, (c) Experiment 2, (d) Experiment 3, and (e) Experiment 4.

To solve the problem that the dataset is too small to train large models, the public large crop pests and diseases dataset Plant-Village is selected for migration learning of VGGNet16. First, the VGGNet16 model is fully trained based on the Plant-Village dataset, and then the number of neurons in the last fully connected layer of the model is modified. The training is continued on the winter wheat pests and diseases dataset. A total of four experimental protocols are designed according to the transfer learning technology, as shown in Figure 6.

In Figure 6, Experiment 1 adopts the transfer learning method of freezing source model, and the parameters of convolution layer (Convs), the first full connection layer (FC_1), and the second full connection layer (FC_2) are copied from the pre-training model. The number of neurons in the third full connection layer (FC_3) is modified to 5 and the parameters are randomly initialized for training. Experiment 2 adopts the transfer learning method of fine-tuning some layers. The parameters of Convs and FC_1 are copied from the pre-training model. After copying the parameters of FC_2, they are fine-tuned in training. The number of neurons in FC_3 is modified to 5 and the parameters are randomly initialized for training. Experiment 3 uses the transfer learning method of fine-tuning part of the layer, and the parameters of the Convs are copied from the pre-training model. After copying the parameters of FC_1 and FC_2, they are fine-tuned in training. The number of neurons in FC_3 is modified to 5 and the parameters are randomly initialized for training. Experiment 4 adopts the transfer learning method of fine-tuning all layers, and the parameters of the Convs are copied from the training model. After the parameters of FC_1 and FC_2 are copied from the pre-training model, they are fine-tuned in training. The number of neurons in FC_3 is modified to 5 and the parameters are randomly initialized for training.

Four transfer learning models are trained based on the expanded training set. To maintain consistency, the hyper-parameter setting of the transfer learning experiment is consistent with VGGNet16. The evaluation indicators of the test set corresponding to the four groups of transfer learning experiments are shown in Table 6.

**Table 6:** VGGNet16 test set evaluation index corresponding to each experimental scheme

Categories	Experiment 1			Experiment 2			Experiment 3			Experiment 4		
	*P*_1_	*R*_1_	*F*_1_	*P*_1_	*R*_1_	*F*_1_	*P*_1_	*R*_1_	*F*_1_	*P*_1_	*R*_1_	*F*_1_
Aphids	0.2718	0.4375	0.3353	0.6225	0.5648	0.5922	0.6667	0.8889	0.7619	0.8182	1.0000	0.9000
Powdery mildew	0.4032	0.2924	0.3390	0.3345	0.2689	0.2981	0.7895	1.0000	0.8824	0.9333	0.9333	0.9333
Healthy	0.6185	0.6917	0.6531	0.8203	0.6192	0.7057	0.8182	0.9310	0.8710	0.9032	0.9655	0.9333
Leaf rust	0.7440	0.6642	0.7018	0.6733	0.7780	0.7219	0.9254	0.8611	0.8921	0.9855	0.9444	0.9645
Stripe rust	0.7093	0.7238	0.7165	0.7519	0.7427	0.7473	0.9429	0.8684	0.9041	0.9867	0.9737	0.9801
Average	0.5494	0.5619	0.5491	0.6405	0.5947	0.6130	0.8285	0.9099	0.8623	0.9254	0.9634	0.9423
*A*_1_	0.6526			0.6937			0.8856			0.9602		

As shown in Table 6, the accuracy of the freezing source model in Experiment 1 is only 65.26%, indicating that the transfer learning method of the freezing source model is not applicable to the dataset in this study. The accuracy of the two fine-tuning partial layers in Experiments 2 and 3 as well as the fine-tuning full layer in Experiment 4 are 69.37, 88.56, and 96.02%, respectively. It can be seen that when more layers are involved in fine-tuning, the better the recognition effect of the model will be. The transfer learning method based on fine-tuning all layers has the best effect.

### 3.4 Improvement based on attention mechanism

**Figure 7:**
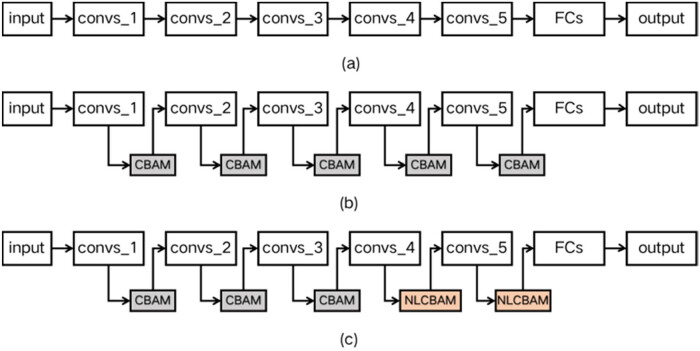
Experimental scheme based on transfer learning. (a) Structural diagram of VGGNet16, (b) structural diagram of CBAM-VGGNet16, and (c) Structural diagram of NLCBAM-VGGNet16.

Since the dataset is sampled in the nutritional growth and reproductive growth stage of wheat, the color and characteristics of wheat pest and disease samples in this stage are quite different and the background of pest and disease samples in natural environment is complex. To this end, the CBAM-VGGNet16 and NLCBAM-VGGNet16 models are further designed. The addition of attention mechanism can enhance the role of relevant features during model training in the channel domain. At the same time, the interference of irrelevant location features on the classification model in the spatial domain can be suppressed. The VGGNet16 network diagram before and after improvement is shown in Figure 7.

In this section, the CBAM-VGGNet16 and NLCBAM-VGGNet16 network models are trained based on the expanded dataset and the experimental parameters are consistent with VGGNet16. The model was evaluated using the identification accuracy (*Single A*_1_) of single pest and disease and the identification accuracy (*A*_1_) of the test sets. In addition, the A-ResNet50 [6] and the Xception-CEMs network model [7] are selected to train based on the expanded dataset for comparison. The experimental results of the final model corresponding to each network model are shown in Table 7.

**Table 7:** Comparison of final model test results

Models	*Single A*_1_					*A*_1_
	Aphids	Powdery mildew	Healthy wheat	Leaf rust	Stripe rust	
A-ResNet50	0.5000	0.6000	0.8000	0.8784	0.9090	0.8398
Xception-CEMs	0.6000	0.6000	0.8333	0.9189	0.9090	0.8640
VGGNet16	0.9000	0.9333	0.9333	0.9595	0.9740	0.9602
CBAM-VGGNet16	0.8000	0.9333	0.9667	0.9730	0.9870	0.9660
NLCBAM-VGGNet16	0.9000	0.9333	0.9667	1.0000	0.9740	0.9757

As shown in Table 7, the recognition accuracies of A-ResNet50 and Xception-CEMs for the test set in the study is only 83.98 and 86.40%, respectively, and the recognition is poor. The recognition effect of VGGNet16 after fine-tuning all layers is better, with 96.02%. Compared with the basic network VGGNet16, CBAM-VGGNet16 has improved the recognition accuracy of all kinds of pests and diseases except powdery mildew. This indicates that the addition of mixed attention mechanism can improve the overall recognition effect of VGGNet16 model. At the same time, compared with VGGNet16, NLCBAM-VGGNet16 has improved the recognition accuracy of all kinds of pests and diseases, and the accuracy can reach 97.57%. Compared with CBAM-VGGNet16, the recognition accuracy of all categories except stripe rust has been improved, indicating that the proposed NLCBAM module can more effectively improve the improvement effect of attention module on the recognition accuracy of network model than CBAM module.

In summary, both these models are improvements based on the attention mechanism of VGGNet16, with varying improvement effects. Compared to CBAM-VGGNet16, NLCBAM-VGGNet16 has a better improvement effect. But the model is also more complex, requiring more time and computational resources during the training process. Therefore, the selection of models should be based on accuracy requirements. If CBAM-VGGNet16 can meet the accuracy requirements, then choose CBAM-VGGNet16. If there are higher requirements, then choose NLCBAM-VGGNet16.

## 4 Conclusion

Aiming at the common pests and diseases of wheat, the dataset of winter wheat pests and diseases is standardized. The VGGNet16 CNN model is trained based on data expansion and migration learning technology, and finally the high-precision identification of common wheat pests and diseases is realized.

1) In the three network models trained based on the improved dataset, VGGNet16 has the highest accuracy and is selected as the basic network for further optimization.
2) The training set is expanded by data expansion technology, so that the number of samples in each category is balanced, and the problem of low recognition accuracy of aphids and powdery mildew in the recognition of VGGNet16 model is solved.
3) The transfer learning experimental scheme of freezing source model, fine-tuning part of layers and fine-tuning all layers is designed. Compared with its recognition accuracy and the *F*1 of each category, the fine-tuning source model is better than the freezing source model. When more layers are involved in fine-tuning, the better the recognition effect of the model will be. The transfer learning method of fine-tuning all layers has the best effect, and the recognition accuracy can reach 96.02%.
4) The recognition effect of CBAM-VGGNet16 and NLCBAM-VGGNet16 based on attention improvement is better than that of VGGNet16. The accuracy rate of the test set of CBAM-VGGNet16 is 96.60%, and that of NLCBAM-VGGNet16 is 97.57%, which realizes the high-precision identification of common pests and diseases of winter wheat.
